# Client Perceptions of the Mental Health Engagement Network: A Secondary Analysis of an Intervention Using Smartphones and Desktop Devices for Individuals Experiencing Mood or Psychotic Disorders in Canada

**DOI:** 10.2196/mental.3926

**Published:** 2015-01-21

**Authors:** Cheryl Forchuk, Lorie Donelle, Paige Ethridge, Laura Warner

**Affiliations:** ^1^ Lawson Health Research Institute London, ON Canada; ^2^ Arthur Labatt Family School of Nursing Faculty of Health Sciences Western University London, ON Canada; ^3^ School of Health Studies Faculty of Science Western University London, ON Canada

**Keywords:** mental health, mobile health, eHealth, personal health records, mood disorders, psychotic disorders, mental disorders

## Abstract

**Background:**

The use of innovative technologies in mental health care has the potential to improve system efficiency, enhance quality of care, and increase patient engagement. The Mental Health Engagement Network (MHEN) project developed, delivered, and evaluated an interactive Web-based personal health record, the Lawson SMART Record (LSR), to assist mental health clients in managing their care and connecting with their care providers. This paper presents a secondary analysis of data collected in the MHEN project regarding clients’ perceptions of technology and the use of these technologies in their care.

**Objective:**

We aimed to answer six questions: (1) What is the level of comfort with technology within a sample of individuals experiencing mood or psychotic disorders? (2) How easy to use and helpful are the MHEN technologies from the perspective of individuals experiencing a mental illness? (3) Are there differences in how helpful or useful individuals find the smartphone compared to the LSR? (4) Are there specific functions of MHEN technologies (eg, reminders for medications or appointments) that are more valued than others? (5) What are the other ways that individuals are using MHEN technologies in their daily lives? (6) How likely are individuals to be able to retain and maintain their smartphone?

**Methods:**

Mental health clients aged 18-80 (N=400) and diagnosed with a mood or psychotic disorder were provided with a smartphone (iPhone 4S) and participating care providers (n=52) were provided with a tablet (iPad) in order to access and engage with the LSR. A delayed implementation design with mixed methods was used. Survey and interview data were collected over the course of 18 months through semistructured interviews conducted by experienced research assistants every 6 months post-implementation of the intervention. Paired *t* tests were used to determine differences between 6 and 12-month data for perceptions of the MHEN technologies. A paired *t* test was used to examine whether differences existed between perceptions of the smartphone and the LSR at 12 months post-implementation.

**Results:**

Due to dropout or loss of contact, 394 out of 400 individuals completed the study. At the end of the study, 52 devices were lost or unusable. Prior to the intervention, participants reported being comfortable using technology. Perceptions of the MHEN technologies and their functions were generally positive. Positive perceptions of the smartphone increased over time (*P*=.002), while positive perceptions of the LSR decreased over time (*P*<.001).

**Conclusions:**

Quantitative and qualitative findings from this analysis demonstrated that these technologies positively impacted the lives of individuals experiencing severe mental illnesses and dispeled some of the myths regarding retention of technology among marginalized populations. This secondary analysis supported the acceptability of using mental health technologies within this population and provided considerations for future development.

**Trial Registration:**

ClinicalTrials.gov NCT01473550; http://clinicaltrials.gov/show/NCT01473550 (Archived by WebCite at http://www.webcitation.org/6SLNcoKb8).

## Introduction

### Background

Health care systems and agencies have increasingly invested in information technology to improve the quality and efficiency of service delivery [1,2]. This trend has extended into mental health care, where the implementation of Electronic Mental Health (e-mental health) has demonstrated positive outcomes [3-5]. The Mental Health Commission of Canada introduced a briefing document outlining the vital role that technology plays in advancing the care of clients within the mental health system [6]. In this document, e-mental health is defined as “mental health services and information delivered or enhanced through the Internet and related technologies” [7]. This broad definition includes the use of diverse technologies such as video conferencing, Web-based interventions, virtual reality platforms, and interventions using mobile devices [6].

The Mental Health Engagement Network (MHEN) used Web and mobile technologies to distribute and evaluate the use of a personal health record (PHR) to assist mental health clients in their care [8-10]. Using a Web-based PHR, an individual can manage and share their health information electronically in a private and secure way. PHRs can be connected to electronic health records (EHRs), which are records of client-related information managed by health care providers. When tethered, EHRs and PHRs create an integrated record of client care that can be used by providers and clients to access information about conditions, medications, test results, and appointments, and to communicate electronically [11]. This paper presents a secondary analysis of data from the MHEN project. The purpose of this secondary analysis is to investigate the perceptions of individuals diagnosed with mental illnesses regarding the use of these technologies in their care.

### Literature Review

Research regarding electronic applications for treatment and maintenance of physical health are increasingly common, but the body of e-mental health work is comparatively less developed [12]. Much of the focus of mental health research using information technology is on the efficacy of specific interventions [13]. For example, studies have shown that mobile and Web-based interventions can effectively deliver cognitive behavioral therapy for depression [14], enhance psychosocial functioning in depressed individuals [15], and decrease psychological distress for individuals experiencing anxiety disorders [16]. As this literature demonstrates, using technology to improve mental health service delivery is promising; however, this is based on assumptions that individuals are comfortable and able to use the technology in regular care. If using technology to manage mental health is unappealing or difficult for clients, the usefulness of these interventions is minimal. Though this literature is growing, research more often focuses on the presence or absence of psychopathology following an intervention, rather than the individuals’ experience using the intervention itself.

Some research, however, is beginning to focus on the use of technology in mental health care from the clients’ perspective [17-20]. Rotondi et al, for example, examined the features of Web-based interventions that enhance usability for mental health clients, including reducing the need to think abstractly or filter out distracting content [20]. Another study conducted online surveys (N=525) to examine community attitudes toward using mobile phones for management of mental health issues [12]. Survey results demonstrated that a majority of participants (76%) were interested in managing mood, anxiety, or health on their mobile devices. Similarly, individuals in the United States living with severe mental illnesses (ie, mood disorders, schizophrenia, or schizoaffective disorder; N=1568) were surveyed regarding their use of mobile technologies and interest in future services. Again, when asked about whether or not they would be interested in receiving mental health services through mobile technologies, 81% of respondents who owned a mobile device and 62% of respondents who did not, responded positively [21]. These findings suggest that technologies can be created so that they are usable by individuals with mental illness and that e-mental health is generally perceived positively by individuals with mental health issues.

However, even clients who express interest in using technology in health management may have difficulty navigating and performing tasks involving technology; thus, it is important to establish the acceptability and usability of technology in addition to client interest [22]. A study that examined an electronic intervention for individuals with schizophrenia or schizoaffective disorder demonstrated that 90% of participants found the intervention to be acceptable and easy to use [23]. Conversely, studies have shown that certain populations may experience significant difficulties in using technology for health management [24]. The roles of cognitive abilities and age in using a simulated PHR for health management activities (eg, health maintenance and medication management) were examined, and the study found that both middle-aged (40-59 years) and older adults (60-85 years) had substantial difficulty in performing health management tasks electronically. Performance was significantly predicted by level of education, Internet experience, cognitive abilities, numeracy skills, and older age [24]. Still, another study found that while individuals with severe mental illness (SMI) and a co-occurring substance use disorder are less likely to use the Internet, there were no significant differences between those who did and did not access the Internet with respect to literacy skills, typing ability, lack of knowledge, or fear of technology. The most common barrier to accessing the Internet was cost [25]. While some research provides evidence suggesting clients perceive an online intervention using a mobile device as an effective way to manage mental health issues, further research into the feasibility of using information technology in mental health care is warranted.

Importantly, none of this research, specifically the studies involving mobile phones, was implemented in Canada. The cost of mobile phone services varies widely between countries, with Canada having some of the highest rates when compared to Australia, the United States, the United Kingdom, France, Germany, Italy, and Japan [26]. Due to this international variation in cost, it is possible that client perceptions regarding use of mobile technology in mental health care may also vary widely between countries. Therefore, further research investigating client perceptions of mobile and Internet-based interventions for mental illness should be carried out in diverse national contexts.

Integrating new technologies into usual health care is dependent on further investigation into what works well for clients and what does not. Research shows that individuals experiencing mental illness are interested in using technology in their mental health care [12]; however, barriers to use and facilitators of adoption of technology in care are not well understood [24,25]. PHRs provide an example of a health care technology that receives significant positive attention while remaining separate from routine service provision. Despite the potential benefits of using PHRs, several barriers to adopting this technology into usual care are apparent: active participation of relevant professionals, data security, cost, and most relevant to this paper, client ability to use the technology [2,11,27,28]. It is not yet fully understood how these barriers influence the use of health care technologies by people with varying levels of cognitive ability, severity of illness, and different geographical locations. It is important, then, to investigate the use of innovative technologies in care with individuals experiencing some of the most severe mental health issues (ie, mood and psychotic disorders) in locations where these services are some of the most expensive in the world (ie, Canada). Findings from such research will significantly contribute to the literature regarding the adoption and use of e-mental health technology in community-based mental health care.

### The Primary Study

The MHEN project sought to deliver and evaluate the use of online resources and mobile technologies in mental health service delivery using a PHR. The project began in September 2011 and was completed in March 2014 in London, Ontario, Canada, and the surrounding area. It used a client-centered intervention designed by an interdisciplinary group of health care providers, researchers, health information technology experts, and mental health clients. Client participants in the project received a smartphone (iPhone 4S), a TELUS health space account, and a Lawson SMART record (LSR). Smartphones were not only communication devices with calling and texting capabilities but also had Internet functionality through data plans and Wi-Fi access. Participating care providers received an LSR account and a tablet (iPad). TELUS health space is powered by Microsoft Health Vault and is a platform on which health information can be gathered, stored, and shared. The LSR is a PHR, that is, a Web-based application, which sits on the TELUS health space platform. Information from EHRs was uploaded on a daily basis to the LSR. This information included an active list of medications, family medical history, immunization records, allergies, mental health care professionals’ contact information, care plans, and crisis plans. The LSR also allowed individuals to input information and included several tools and functionalities: a mood monitor to track, store, and share moods with their participating health care professional; health journal notes to log subjective thoughts and reminders; prompts and reminders to assist in daily living; the ability to track physiological measures (eg, blood pressure, blood glucose, weight); and secure messaging with their mental health care professional. The intervention as well as its adoption by clients and providers has previously been reported in greater detail [8-10].

### The Study

This study is a secondary analysis of data from the MHEN project, which assessed client participants’ perceptions regarding use of the MHEN technologies (ie, the smartphone and the LSR). This investigation will elaborate on the little that is known about how individuals experiencing mental illness use health care technology. It is necessary to assess factors that influence client adoption so that the implementation of health care technologies can be feasible. In order to further understand factors affecting the use of technology in mental health care, this study addressed several research questions:

1. What is the level of comfort with technology within a sample of individuals experiencing mood or psychotic disorders?
2. How easy to use and helpful are the MHEN technologies from the perspective of individuals experiencing a mental illness?
3. Are there differences in how helpful or useful individuals find the smartphone compared to the LSR?
4. Are there specific functions of the MHEN technologies (eg, prompts and reminders for medications or appointments, being able to connect with their care provider, ability to share information with other providers) that are more valued than others?
5. What are the other ways these individuals are using the MHEN technologies in their daily lives?
6. How likely are individuals to be able to retain and maintain their phone (eg, lose or break it)?

This is an essential initiative given the current emphasis on developing information technology to enhance health care service delivery and the potential benefits for consumers of using these technologies in regular care [6].

## Methods

### Design

The current study is a secondary analysis of the information obtained through Demographics and Perception of SMART Technology questionnaires, both of which were designed by the research team. Data collected through these forms was used to assess a baseline comfort with technology and feelings towards the technologies used in the MHEN project.

The MHEN project was based on a delayed implementation design and employed a mixed methods approach. Community-based individuals from the caseloads of participating mental health care professionals were randomized into two groups: individuals in Group A (early intervention group) received the smart technology intervention first, while those in Group B (delayed intervention group) acted as a control for the first 6 months and thus received the intervention 6 months after Group A. Surveys were used to assess demographics, empowerment, health status, health and social services use, quality of life, and perceptions of SMART technology. Experienced research assistants administered questionnaires every 6 months for a total of 18 months, resulting in four interview points. Interviews occurred in a location of the client’s choosing, including the research office, the individual’s home, or a community setting such as a coffee shop. Qualitative data were obtained through focus group sessions that occurred throughout the study, in addition to open-ended questions answered during the survey administration. Maximum variation sampling was used to identify potential participants for focus groups.

Individuals in the study received CAN $20 for their participation in each interview and focus group. Participants gave informed consent prior to receiving the intervention and before each interview. Ethics approval was obtained from the university research ethics board in December 2011. See Multimedia Appendix 1 for the CONSORT E-HEALTH checklist [29].

### Sample

In total, 400 community-based participants were recruited from the caseloads of 54 mental health care professionals in London, Ontario, and the surrounding area. The health care providers were members of one of four community mental health agencies. Participating care providers asked clients on their caseloads if they would be interested in participating in the study. Interested clients contacted the research team directly to indicate their interest and schedule a time for registration. Eligible clients were between the ages of 18 and 80, had been diagnosed with either a mood or psychotic disorder, and were able to read and understand English. As a result of dropouts and loss of contact with participants, the analysis presented here is based on 394 individuals who completed the study. Group A consisted of 192 individuals, and Group B consisted of 202 individuals.

### Measures

Assessment focused on participants’ perceptions and self-reports of their experiences using the software, smartphone, and desktop computer interfaces in the context of the project. Usability testing of the devices or software was not conducted.

Baseline level of comfort with technology was assessed through three questions asking how comfortable the participant felt with computers, the smartphone, and technology generally. Responses ranged from 1 (extremely comfortable) to 7 (extremely uncomfortable). As the more extreme categories contained fewer individuals than the more central categories, responses were collapsed into three categories: comfortable, mixed, and uncomfortable.

Participants were asked to think only of their smartphone without their health record and indicate how easy it was to use, how helpful it was, how simple it was to use, and how much independence it afforded. They were then asked to think only of the LSR and indicate the same. Initially, responses were scored from 1 to 7, with 1 representing negative feelings in some cases (ie, extremely hard to use, extremely unhelpful) and positive in other cases (ie, extremely simple to use, gives extremely more independence). For the analysis, these were rescored so 1 represented extremely negative feelings (ie, hard to use, unhelpful, confusing, less independence) and 7 represented extremely positive feelings (ie, easy to use, helpful, simple, more independence).

Individuals were asked to indicate how they felt about each specific feature of the smartphone and health record on a scale from 1 (terrible) to 7 (delighted). These features included having their own PHR, receiving medication prompts, receiving appointment/schedule prompts, connecting with their care provider using the smartphone, connecting with their care provider using the LSR, having access to their personal crisis plan, and being able to share their health information with other health care providers.

Participants were asked specifically whether or not they used the LSR, and whether or not they used the smartphone in order to determine utilization rates. They were also asked to indicate what they used the smartphone for. The list of possible uses included accessing the LSR, contacting their care provider, use of social media (eg, Facebook, Twitter), texting, emailing, playing games, listening to music, watching videos, or other. If they indicated “other”, they were asked to give specific details of use. For the current analysis, these details were examined and additional categories created. Each participant could indicate multiple items.

Most items from the Perception of SMART Technology questionnaire were collected at the 6, 12, and 18-month interviews. The exceptions were the questions on feelings about connecting with their care provider through their health record, questions on utilization of the smartphone and LSR, and what participants were using their smartphones for. This information was only collected at the 12 and 18-month interviews.

### Analysis

**Figure 1:**
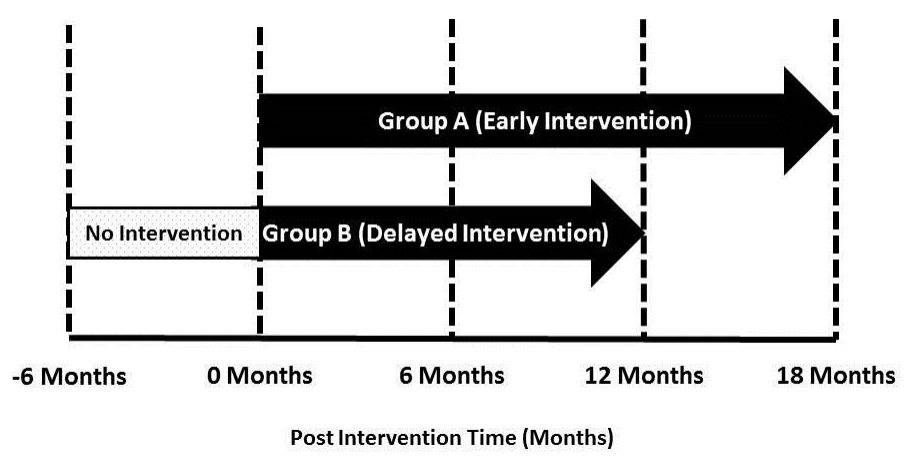
Study design for the MHEN intervention identifying post-intervention and post-implementation time points for each treatment group.

As there were no significant differences between the two intervention groups post-randomization, they were collapsed into one group for purposes of the current analysis. To account for the delayed implementation design, data were analyzed from an intervention time perspective instead of an interview time perspective. To achieve this, data from Group A (early intervention group) remained unchanged while data from Group B (delayed intervention group) were shifted back (Figure 1).

Frequencies and percentages were calculated for all categorical data (eg, sample characteristics, baseline comfort with technology, utilization of the smart technologies), and means and standard deviations were calculated for all scale variables (eg, feelings towards the technologies in general and the specific features of each).

Paired *t* tests were used to determine differences between 6 and 12-month post-intervention data for perceptions of the smartphone and LSR regarding ease of use, helpfulness, simplicity, and independence they afforded. Perceptions about specific features of the smartphone and LSR were also compared. Additionally, a paired *t* test was used to examine whether differences existed between perceptions of the smartphone and the health record at 12 months post-intervention. Each specific analysis was conducted on a complete case basis, and all data analyses were done using Statistical Package for Social Sciences (SPSS) 22.0.

Qualitative data from focus groups and open-ended questions were thematically analyzed, and supporting quotations were captured to further understand quantitative results. Focus group discussions were audio recorded and subsequently transcribed verbatim. Transcripts were read by the research assistant and principal investigator, and themes were established using a matrix approach [30]. Analysis was guided by Leininger’s approach to qualitative analysis [31]. Discussion of initial themes occurred at team meetings, and transcripts were subsequently re-read in order to further refine themes.

## Results

### Sample Characteristics

The average age of MHEN participants was 37.6 years, and the majority of participants were male (239/394, 60.7%) and/or single and had never been married (276/394, 70.1%). Just under half (177/394, 45.0%) of the individuals in the study had graduated high school, and almost a quarter (97/394, 24.6%) had completed post-secondary schooling. The most prevalent psychiatric diagnosis in the sample was a psychotic disorder (234/394, 59.4%) followed closely by a mood disorder (226/394, 57.4%). The least prevalent diagnoses were personality disorder (24/394, 6.1%), disorder of childhood/adolescence (22/394, 5.6%), and other/organic/unknown type (19/349, 4.8%). No significant differences between the early intervention group and delayed intervention were found on any baseline demographics (Table 1).

**Table 1:** Sample characteristics (N=394) of MHEN study participants.

Characteristics		Early intervention group, n (%)	Delayed intervention group, n (%)	Total sample, n (%)
Age in years, mean (SD)		38.2 (14.6)	37.1 (12.9)	37.6 (13.8)
**Sex**				
	Male	125 (65.1)	114 (56.4)	239 (60.7)
	Female	67 (34.9)	88 (43.6)	155 (39.3)
**Marital status**				
	Single, never married	140 (72.9)	136 (67.3)	276 (70.1)
	Married/Common-law	18 (9.4)	15 (7.4)	33 (8.4)
	Separated/Divorced	32 (16.7)	50 (24.8)	82 (20.8)
	Widowed	2 (1.0)	1 (0.5)	3 (0.8)
**Highest level of education**				
	Grade school	52 (27.1)	66 (32.8)	119 (30.3)
	High school	93 (48.4)	84 (41.8)	177 (45.0)
	Community college/ University	46 (24.0)	51 (25.4)	97 (24.6)
Currently employed		47 (24.5)	50 (24.8)	97 (24.6)
**Psychiatric diagnoses** ^a^				
	Psychotic disorder	111 (57.8)	123 (60.9)	234 (59.4)
	Mood disorder	119 (62.0)	107 (53.0)	226 (57.4)
	Anxiety disorder	60 (31.2)	64 (31.7)	124 (31.5)
	Substance related disorder	30 (15.6)	20 (9.9)	50 (12.7)
	Personality disorder	12 (6.2)	12 (5.9)	24 (6.1)
	Disorder of childhood/ adolescence	9 (4.7)	13 (6.4)	22 (5.6)
	Other/organic/unknown	8 (4.0)	11 (5.5)	19 (4.8)

^a^Individuals could have multiple diagnoses and therefore be counted in more than one group. Diagnosis groups do not add to 100%.

### Comfort With Technology Prior to Intervention

**Figure 2:**
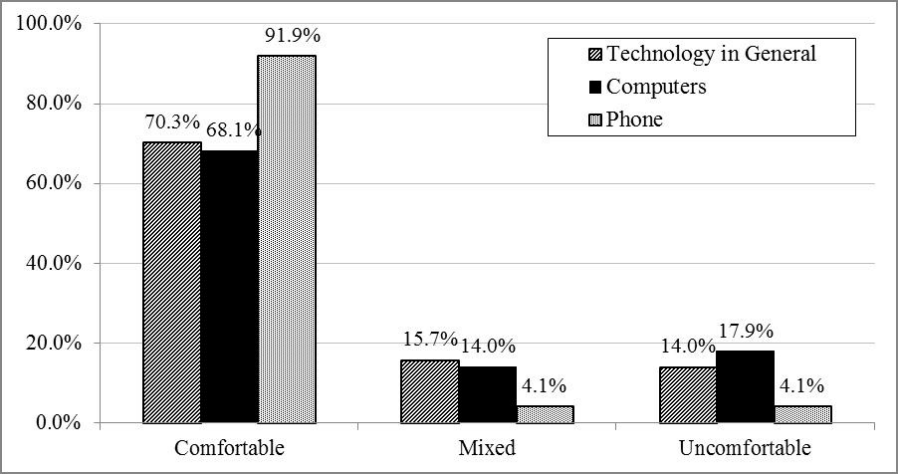
Level of comfort with technology in general, computers, and phones at baseline.

At the outset of the study, the majority of participants felt comfortable with all of the technologies that were investigated (computers, phone, and technology in general; Figure 2). Almost the entire sample (362/394, 91.9%) felt comfortable with phones, and approximately two-thirds felt comfortable with computers (267/394, 68.2%) and technology in general (277/394, 70.3%). Alternatively, only a small fraction (16/394, 4.1%) felt uncomfortable with phones, and less than one-fifth of the sample felt uncomfortable with computers (70/392, 17.9%) or technology in general (55/394, 14.0%).

### Perceptions of the Smartphone and Lawson SMART Record

Given a neutral score of four (half way between the extreme negative and positive scores), it appears perceptions of ease of use, helpfulness, simplicity, and provision of independence for both the smartphone and the LSR were generally positive as all averaged scores ranged from 4.83 to 6.29 (Table 2). Only two aspects significantly changed over time: positive perceptions towards the smartphone’s ease of use increased between 6 and 12 months post-intervention (*t*_311_=-3.112, *P*=.002), while positive perceptions towards the LSR’s helpfulness decreased (*t*_219_=4.443, *P*<.001).

**Table 2:** Client perception scores regarding ease of use, helpfulness, simplicity, and provision of independence of the smartphone and Lawson SMART Record over time.

		n	6 months post-intervention	12 months post-intervention	18 months post-intervention^a^	*P* ^b^
**Smartphone**						
	Ease of use	312	5.32 (1.71)	5.61 (1.67)	5.65 (1.68)	.002
	Helpfulness	310	6.29 (1.05)	6.28 (1.11)	6.24 (1.09)	.93
	Simplicity	311	5.26 (1.80)	5.24 (1.80)	5.27 (1.82)	.83
	Independence	310	5.84 (1.32)	5.76 (1.48)	5.84 (1.49)	.41
**Lawson SMART Record**						
	Ease of use	218	4.91 (1.87)	4.83 (1.94)	4.92 (1.84)	.55
	Helpfulness	220	5.80 (1.11)	5.35 (1.60)	5.15 (1.59)	<.001
	Simplicity	220	5.00 (1.75)	4.88 (1.79)	5.07 (1.70)	.36
	Independence	221	5.26 (1.43)	5.10 (1.46)	5.16 (1.35)	.14

^a^Data from Group A only. Due to the delayed implementation design Group B did not have 18-month post-intervention data.

^b^ *P* value reflects difference between 6 and 12 months post-intervention.

These findings mirror the qualitative data from the focus groups. With respect to ease of use of the phone, a number of individuals mentioned how much easier they were finding the phone to use over time:

I’m able to use the phone a little bit easier than I could at first…because I’m learning more about certain apps that I have and if I think of something, the people in my family that are more tech savvy, I can get them to help me… it was new for me when I first got it, but more and more as I kind of work on my phone, ’cause I got a few games that I’m learning to use the apps a little bit easier.

Well um, I know how to text now, I know how to um use the smartphone itself and umm how to dial the numbers that I need and just push a button and you can find what number you want and just contacts. Yeah, it’s a lot easier now.

One of the other themes that emerged through open-ended questions, in support of the quantitative findings above, was the decrease in use of the smart record over time due to an improved mental health status, or recovery. This could explain why individuals were finding the record less useful over time. For example, when asked at the 12-month post-intervention point whether they still used the LSR, one participant noted “I’m not really using it right now. I’m doing well right now and so I don’t want to be reminded of my mental health problems,” and another stated, “I haven’t had any mental health issues lately. So I haven’t had a need to use it”. At 18 months post-intervention, another participant stated they were not using the smart record because “I kind of became more stable since the study began so it didn’t seem as necessary.”

When comparing the smartphone and LSR at 12 months post-intervention, individuals consistently rated the smartphone higher than the LSR. The difference in average scores between the two technologies ranged from 0.56 (simplicity) to 1.01 (ease of use), depending on the utility being examined, and were all significant (all *P*<.001; Table 3).

Some of the best examples in the difference between perceptions of the smartphone and LSR come from the medication and appointment prompts and reminders. Individuals often felt the phone outperformed the record in reliability and helpfulness and subsequently began using the applications native to the phone for reminders and alarms in place of the record’s prompts:

Um, I had some problems with the TELUS health space part because I would enter times for medication and instead of sending one email it would send me three or four. So if you’re taking medication a couple times a day, that’s almost a dozen emails. Um, so I stopped using that part of the TELUS and I’ve programmed it into the reminders and alerts on the phone.

I find that, unfortunately, I rely more on the calendar for reminders than the [Lawson SMART Record] app because they are very correct, like it’s 7, it’s coming on at 7.

**Table 3:** Scores for comparison of feelings towards the smartphone and Lawson SMART Record regarding ease of use, helpfulness, simplicity, and independence they afford at 12 months post-intervention.

	Smartphone	Lawson SMART Record	*P* value
Ease of use	5.71 (1.64)	4.70 (1.98)	<.001
Helpfulness	6.24 (1.17)	5.33 (1.60)	<.001
Simplicity	5.33 (1.76)	4.77 (1.86)	<.001
Independence	5.72 (1.49)	5.05 (1.46)	<.001

### Value of the Smartphone and Lawson SMART Record Functions

Again, perceptions of specific functions of the smartphone and the LSR tended to be positive overall, with scores ranging from 4.97 to 5.90 (Table 4). Over time there was a significant decrease in the positivity of perceptions towards having the LSR (difference of 0.24, *P*=.002) and having access to a personal crisis plan (difference of 0.29, *P*=.009). No other significant changes were found.

**Table 4:** Scores for perceptions about specific functions of the smartphone and Lawson SMART Record over time.

	n	6 months post-intervention	12 months post-intervention	18 months post-intervention^a^	*P* ^b^
Having own PHR	286	5.76 (1.09)	5.52 (1.23)	5.41 (1.27)	.002
Medication prompts	44	5.50 (1.41)	5.57 (1.17)	5.89 (0.60)	.78
Appointment/Schedule prompts	95	5.64 (1.25)	5.66 (1.32)	5.67 (1.18)	.89
Connecting with care provider using smartphone	250	5.75 (1.20)	5.65 (1.29)	5.45 (1.63)	.31
Connecting with care provider using PHR	90	5.34 (1.52)^c^	5.14 (1.43)^c^	4.97 (1.69)	.20^c^
Having access to personal crisis plan	119	5.90 (0.97)	5.61 (1.14)	5.45 (1.42)	.009
Ability to share health information with other care providers	270	5.65 (1.21)	5.54 (1.25)	5.35 (1.33)	.24

^a^Data from Group A only. Due to the delayed implementation design, Group B did not have 18-month post-intervention data.

^b^ *P* value reflects difference between 6 and 12 months post-intervention.

^c^Reflects Group B only due to design and timing of questionnaire.

### Use of the Smartphone and Lawson SMART Record

At 12 months post-intervention, 311 (93.4%) of the participants who answered questions (N=335) about use of the smartphone and PHR indicated they were currently using the smartphone. In contrast, 151 (45.3%) indicated they were currently using the LSR. The five most common uses of the smartphone were related to communication (Table 5). Of those who indicated they were currently using the smartphone, 247 (79.4%) indicated they were using it to send and receive text messages, 240 (77.2%) indicated they were using it to contact their care provider, and 205 (65.9%) indicated they were using it to send and receive email messages. Accessing the LSR was the seventh most common activity with 145 (46.6%) of those reporting on the use of the smartphone.

From the focus group data, the theme of being able to reach out and contact someone was identified: “I can text my sister in [British Columbia]and she can phone me back…it makes me feel better that I can actually use something like type something and she can receive it. It gives me more confidence”, “Yeah actually, I text. I text my older brother or call my friends, texting away, call them, keeps me in contact with everybody”, and:

Also just, like, the phone is right there like, if I really want to talk to somebody it’s a lot easier for me, instead of writing down, to just go and call my worker, or call support, or call a crisis line. It just seems a lot easier. Or you know, um texting people if I’m in crisis, I’ll text my step daughter or my mom.

**Table 5:** Most common uses of the smartphone.

Rank	Uses	12 months post-intervention, n (%)
1	Texting	247 (79.4)
2	Contacting care provider	240 (77.2)
3	Listening to music	210 (67.5)
4	Email	205 (65.9)
5	Watching videos	174 (55.9)
6	Social media	166 (53.4)
7	Accessing the Lawson SMART Record	145 (46.6)
8	Games	141 (45.3)
9	Social phone calls/Communication	35 (11.3)
10	Internet browsing	20 (6.4)
11	Alarms/Calendar	16 (5.1)
12	Camera/Photography	13 (4.2)
13	Checking weather	10 (3.2)
14	Other^a^	9 (2.9)
15	Banking	6 (1.9)
16	Reading/Studying	6 (1.9)
17	Notes	5 (1.6)
18	Apps in general	5 (1.6)
19	GPS/Maps	3 (1.0)

^a^Includes unspecified, making/recording music, guitar tuning, checking stocks, job searching, organization.

### Maintenance of Smartphones

At the study’s completion, a total of 62 devices had been lost, sold, broken, stolen, or permanently locked at some point during the study. Of the 30 devices that had been lost, 10 were later found by participants, resulting in a total of 52 unusable or misplaced devices. Of these, 20 (38.5%) had been lost, 17 (32.7%) had been stolen, 8 (15.3%) had been broken, 6 (11.5%) had been sold, and 1 (1.9%) had been permanently locked due to the security features of the operating system.

## Discussion

### Principal Results

The MHEN project used mobile phones and Web-based technologies to support the care of individuals experiencing mental illness [8-10]. The purpose of this secondary analysis was to determine how participants in the study perceived the use of the smartphone and the PHR in their mental health care and in their lives generally. There is early evidence supporting the notion that mobile and Web-based technologies are viable methods of improving mental health care service delivery and research [32-34]; yet, little is known about how adults with mood and psychotic disorders use technology in their lives or their ability to use these technologies in their care. This secondary analysis of data from the MHEN project provided insight into client perceptions of these technologies and supported the feasibility of implementing similar technologies into the wider system of mental health care.

At baseline, individuals indicated that they were generally comfortable using technology. This is an important finding given the severity of illness experienced by the research participants; for example, individuals in this study had an average of 7-8 psychiatric hospitalizations in their lifetimes. These findings suggest that the decreased likelihood of owning a mobile device and using the Internet for those diagnosed with mental illnesses, as compared to the general population [21,35], is not likely due to discomfort in using technology. Perhaps, given the resources required to own and use mobile devices, the digital divide between those experiencing mental illness and the general population would disappear. Further, these initial ratings of comfort with technology are consistent with previous research [36] and support the feasibility of implementing new technologies into the care of those with SMIs.

When evaluating the use of technologies in mental health care, it is important to consider perceptions of the specific technologies involved. Participants perceived the LSR and mobile devices used in this intervention positively in terms of ease of use, helpfulness, simplicity, and the independence they afforded. Interestingly, the positivity of perceptions of the smartphone increased over time, while the positivity of perceptions of the LSR decreased over time. The increase in participants’ positive perceptions of the smartphone over time could be a result of increased familiarity with the device and ability to navigate its functions. This finding supports the idea that individuals with SMIs are able to learn and adapt to using complex technologies. The decrease in participants’ positive perceptions of the LSR over time may be indicative of an improvement in mental health status over the course of the study. If participants’ symptoms of mental illness were improving and they felt less in need of intensive treatment, it follows that the LSR would be perceived as being less helpful over time. Another possible explanation of this decrease in LSR use could be related to issues of the LSR’s functionality. An onerous login process, for example, could have increasingly deterred clients from accessing the LSR over time. Both explanations correspond to participants consistently rating the smartphone more positively than the PHR at the conclusion of the study.

Overall, participants rated specific functions of the technologies, such as appointment reminders, as being perceived positively. Participants’ perceptions of having a PHR and having access to a personal crisis plan decreased over time. Other functions, such as medication and appointment reminders, proved to be important to many participants. However, participants often found these functions within the LSR to be lacking and consequently used functions native to the smartphone, such as the calendar, for these purposes. These findings suggest that while the smartphone and its functions appear to be helpful, the LSR will benefit from further modification. In addition, these findings point to the programming capabilities of individuals who are experiencing SMIs as evidenced by participant modification of the smartphone functions to meet their individual care needs.

Consistent with previous research findings [12], participants most commonly reported using the smartphone for communication. Many individuals suggested that a prominent benefit of the intervention was simply being able to stay connected with friends and family and easily contact a care provider when in crisis. Sending and receiving text messages were reported as the most common uses of the phone. Future interventions should be developed with this observation in mind and leverage client comfort with text-based communication.

A concern at the outset of the study was maintenance and retention of devices. The small percentage of devices that were lost, stolen, broken, or inactive at the completion of the study provides evidence that individuals diagnosed with SMIs are accountable to manage and maintain devices for personal and health management purposes. With the aim of integrating mental health technologies into usual care, it is important to consider the feasibility of providing large numbers of individuals with costly devices. Even if only 13% of clients are unable to retain or maintain their devices, as was found in the present study, replacing these devices would be a substantial additional cost to service providers. For this reason, future research should investigate the use of interventions that do not require the use of costly smartphones with data plans, such as those that are text message–based.

### Limitations

Several limitations of this research should be considered when evaluating client perceptions of this intervention and when planning similar interventions in the future. For example, since the LSR is often accessed using the smartphone, it is difficult to evaluate the phone and the record as separate entities. While questions about the phone were separated from questions about the LSR, clients may not have completely separated the two components in their perceptions. Additionally, the LSR is available in both desktop computer and mobile phone versions, and so it is possible that clients were using their smartphones to access the version of the LSR designed for use on desktop computers. It is important to understand how individuals prefer to access their information and what characteristics make a function appealing and usable. Fully understanding user preferences may not be possible without knowing the way that individuals accessed the LSR, but this was not probed in this study. Another possible limitation is the link between a client’s perception of the intervention and their care provider’s willingness or ability to use the intervention. The intervention examined in the MHEN study involved two-way input from clients and care providers. In order for clients to perceive the intervention positively, care providers must be able to support clients in the technical aspects of the intervention and also be actively engaged in the intervention themselves [37]. Unfortunately, active care provider participation was not always present and for this reason, some clients may have perceived the LSR negatively. These factors should be considered in the design and implementation of future research in order to assist in understanding the perceptions of individuals experiencing mental illness regarding the use of smartphones, desktop computers, and health management software in their mental health care.

### Conclusions

The implementation of innovative technologies to improve the quality and efficiency of care for individuals experiencing mental illness is a promising avenue for system improvement. Though future research is needed to elaborate on factors that make technological interventions acceptable, usable, and cost-effective, this secondary analysis of data from an e-mental health intervention study has provided evidence supporting the applicability of mental health technologies in the care of individuals with severe mental illnesses.

## Appendix

Multimedia Appendix 1 CONSORT-EHEALTH checklist V1.6.2 [37].

## Abbreviations

EHR: electronic health record
LSR: Lawson SMART Record
MHEN: Mental Health Engagement Network
PHR: personal health record
SMI: severe mental illness
