# Supplementary material for: Client Perceptions of the Mental Health Engagement Network: A Secondary Analysis of an Intervention Using Smartphones and Desktop Devices for Individuals Experiencing Mood or Psychotic Disorders in Canada
Source: JMIR Ment Health. 2015 Jan 21;2(1):e1. doi: 10.2196/mental.3926 (PMC4607388; doi:10.2196/mental.3926)
Supplement: Multimedia Appendix 1 [file mental_v2i1e1_app1.pdf]

CONSORT-EHEALTH Checklist V1.6.2 Report

(based on CONSORT-EHEALTH V1.6), available at [http://tinyurl.com/consort-ehealth-v1-6].

3926

Date completed

10/7/2014 12:12:32

by

Cheryl Forchuk

Client perceptions of the Mental Health Engagement Network: A secondary analysis of a mobile and web-based intervention for individuals experiencing severe mental illness

TITLE

1a-i) Identify the mode of delivery in the title

"A secondary analysis of a mobile and web-based intervention for individuals experiencing severe mental illness "

1a-ii) Non-web-based components or important co-interventions in title

1a-iii) Primary condition or target group in the title

"for individuals experiencing severe mental illness"

ABSTRACT

1b-i) Key features/functionalities/components of the intervention and comparator in the METHODS section of the ABSTRACT

"Mentalhealth clients aged 18 to 80 (n = 400) and diagnosed with a mood or psychotic disorder were provided with a smartphone (iPhone 4S) and participating care providers (n = 52) were provided with a tablet (iPad) in order to access and engage with the [Lawson SMART Record]. A delayed implementation design with mixed methods was used. Survey and interview data was collected over the course of 18 months through semi-structured interviews conducted every six months post-implementation of the intervention."

1b-ii) Level of human involvement in the METHODS section of the ABSTRACT

"...and participating care providers (n = 52) were provided with a tablet (iPad) in order to access and engage with the [Lawson SMART Record]."

1b-iii) Open vs. closed, web-based (self-assessment) vs. face-to-face assessments in the METHODS section of the ABSTRACT

"Survey and interview data was collected over the course of 18 months through semi-structured interviews conducted by experienced research assistants every six months post-implementation of the intervention."

1b-iv) RESULTS section in abstract must contain use data

"Due to drop out or loss of contact, 394 out of 400 individuals completed the study. At the end of the study 52 devices were lost or unusable."

This study involves a secondary analysis to assess clients' perceptions of using the technologies provided. Web metrics and use data (e.g., number of logins) have been reported in previous publications.

1b-v) CONCLUSIONS/DISCUSSION in abstract for negative trials

"Quantitative and qualitative findings from this analysis demonstrated that these technologies positively impacted the lives of individuals experiencing severe mental illnesses and dispelled some of the myths regarding retention of technology among marginalized populations. This secondary analysis supported the acceptability and applicability of using mental health technologies within this population and provided considerations for future development."

INTRODUCTION

2a-i) Problem and the type of system/solution

"Healthcare systems and agencies have increasingly invested in information technology to improve the quality and efficiency of service delivery...This paper will present a secondary analysis of data from the MHEN project. The purpose of this secondary analysis is to investigate the perceptions of individuals diagnosed with mental illnesses regarding the use of these technologies in their care... The MHEN project sought to deliver and evaluate the use of online resources and mobile technologies in mental health service delivery using a [Personal Health Record]."

2a-ii) Scientific background, rationale: What is known about the (type of) system

The study was designed by a range of key stake holders including researchers, clinicians, and individuals with lived experience of mental illness. The main purpose of this study was to understand the use of these technologies from the perspective of mental health clients.

"Healthcare systems and agencies have increasingly invested in information technology to improve the quality and efficiency of service delivery [1, 2]. This trend has extended into mental health care in which the implementation of Electronic Mental Health (e-mental health) has demonstrated positive outcomes [3-5]. The Mental Health Commission of Canada recently introduced a briefing document outlining the vital role that technology plays in advancing the care of clients within the mental health system [6]."

"Several similar technologies have been investigated. While some interventions have proven effective, results related to the usability of technology in mental health care are mixed: "A study that examined an electronic intervention for individuals with schizophrenia or schizoaffective disorder demonstrated that 90% of participants found the intervention to be acceptable and easy to use [24]. Conversely, studies have shown that certain populations may experience significant difficulties in using technology for health management [25]. The roles of cognitive abilities and age in using a simulated PHR for health management activities (e.g., health maintenance, lab/test results, and medication management) were examined and the study found that both middle-aged (40-59 years) and older adults (60-85 years) had substantial difficulty in performing health management tasks electronically. Performance was significantly predicted by level of education, internet experience, cognitive abilities, numeracy skill, and older age [25]."

"Integrating new technologies into usual health care is dependent on further investigation into what works well for clients and what does not...Findings from such research will significantly contribute to the literature regarding the adoption and use of e-mental health technology in community-based mental health care."

METHODS

3a) CONSORT: Description of trial design (such as parallel, factorial) including allocation ratio

"In order to further understand factors affecting the use of technology in mental health care this study addressed several research questions:

1. What is the level of comfort with technology within a sample of individuals experiencing mood or psychotic disorders?
2. How easy to use and helpful are the MHEN technologies from the perspective of individuals experiencing a mental illness?
3. Are there differences in how helpful or useful individuals find the smartphone compared to the LSR?
4. Are there specific functions of the MHEN technologies (e.g., prompts and reminders for medications or appointments, being able to connect with their care provider, ability to share information with other providers) that are more valued than others?
5. What are the other ways these individuals are using the MHEN technologies in their daily lives?
6. How likely are individuals to be able to retain and maintain their phone (e.g., lose or break it)?"

3b) CONSORT: Important changes to methods after trial commencement (such as eligibility criteria), with reasons

No important changes were made to the methods after trial commencement.

3b-i) Bug fixes, Downtimes, Content Changes

Minor changes were made to the LSR after Group A gave initial feedback on the system's usability; for instance, "End Date" for diagnoses was added in the case that a client was no longer experiencing a particular issue. No major changes in content were made.

4a) CONSORT: Eligibility criteria for participants

"In total, 400 community based participants were recruited from the caseloads of 54 mental health care professionals in London, Ontario and the surrounding area. The health care professionals were members of four community mental health agencies including London Health Sciences Center, St. Joseph’s Health Care (London and St. Thomas), the Canadian Mental Health Association, and WOTCH Community Mental Health Care Services. Individuals were between the ages of 18 and 80, had been diagnosed with either a mood or psychotic disorder, and were able to read and understand English."

4a-i) Computer / Internet literacy

Computer/Internet literacy was not an eligibility criterion. Training was provided to clients prior to being given the intervention on how to use the technology and drop-in sessions were available for clients to attend if they had specific questions or difficulties using the technology.

4a-ii) Open vs. closed, web-based vs. face-to-face assessments:

"In total, 400 community based participants were recruited from the caseloads of 54 mental health care professionals in London, Ontario and the surrounding area."

"Experienced research assistants administered questionnaires every six months for a total of 18 months, resulting in four interview points... Qualitative data was obtained through [in person] focus group sessions that occurred throughout the study, in addition to open ended questions answered during the survey administration."

4a-iii) Information giving during recruitment

4b) CONSORT: Settings and locations where the data were collected

"Interviews occurred in a location of the client's choosing, including the research office, the individual's home, or a community setting such as a coffee shop."

4b-i) Report if outcomes were (self-)assessed through online questionnaires

"Surveys were used to assess demographics, empowerment, health status, health and social services use, quality of life, and perceptions of SMART technology. Experienced research assistants administered questionnaires every six months for a total of 18 months, resulting in four interview points."

Measures were self-report but were administered by research staff in person.

4b-ii) Report how institutional affiliations are displayed

5) CONSORT: Describe the interventions for each group with sufficient details to allow replication, including how and when they were actually administered

5-i) Mention names, credential, affiliations of the developers, sponsors, and owners

5-ii) Describe the history/development process

5-iii) Revisions and updating

5-iv) Quality assurance methods

5-v) Ensure replicability by publishing the source code, and/or providing screenshots/screen-capture video, and/or providing flowcharts of the algorithms used

5-vi) Digital preservation

5-vii) Access

"Client participants in the project received a smartphone (iPhone 4S), a TELUS health spaceTM account, and a Lawson SMART record (LSR). Smartphones were not only communication devices with calling and texting capabilities, but also had internet functionality through data plans and Wi-Fi access. Participating care providers received a LSR account and a tablet (iPad)."

Participants were not required to pay for their smartphone/tablet, phone plans or data services. Phone plans and data services were provided for the duration of the study, and participants were able to keep devices free of charge after the study ended.

5-viii) Mode of delivery, features/functionalities/components of the intervention and comparator, and the theoretical framework

"TELUS health spaceTM is powered by MicrosoftTM Health VaultTM and is a platform on which health information can be gathered, stored and shared. The LSR is a PHR, a web-based application, which sits on the TELUS health spaceTM platform. Information from EHRs was uploaded on a daily basis to the LSR. This information included an active list of medications, family medical history, immunization records, allergies, mental health care professionals' contact information, care plans, and crisis plans. The LSR also allowed individuals to input information and included several tools and functionalities: a mood monitor to track, store, and share moods with their participating health care professional; health journal notes to log subjective thoughts and reminders; prompts and reminders to assist in daily living; the ability to track physiological measures (e.g., blood pressure, blood glucose, weight); and secure messaging with their mental health care professional. The intervention as well as its adoption by clients and providers has previously been reported in greater detail [8-10]."

5-ix) Describe use parameters

5-x) Clarify the level of human involvement

5-xi) Report any prompts/reminders used

Prompts and reminders were not used to encourage use of the application.
5-xii) Describe any co-interventions (incl. training/support)
As this paper is reporting a secondary analysis focused on client perceptions of the technology, detailed information about training was not included. This information has been previously reported elsewhere.
6a) CONSORT: Completely defined pre-specified primary and secondary outcome measures, including how and when they were assessed
"The current study is a secondary analysis of the information obtained through demographics and Perception of SMART Technology questionnaires, both of which were designed by the research team. Data collected through these forms was used to assess a baseline comfort with technology and feelings towards the technologies used in the MHEN project."

"Baseline level of comfort with technology was assessed through three questions asking how comfortable the participant felt with computers, the phone, and technology generally. Responses ranged from 1 (extremely comfortable) to 7 (extremely uncomfortable). As the more extreme categories contained fewer individuals than the more central categories, responses were collapsed into three categories: comfortable, mixed, and uncomfortable.

Participants were asked to think only of their smartphone without their health record and indicate how easy it was to use, how helpful it was, how simple it was to use, and how much independence it afforded. They were then asked to think only of the LSR and indicate the same. Initially, responses were scored from 1 to 7, with 1 representing negative feelings in some cases (i.e., extremely hard to use, extremely unhelpful) and positive in other cases (i.e., extremely simple to use, gives extremely more independence). For the analysis these were rescored so 1 represented extremely negative feelings (i.e., hard to use, unhelpful, confusing, less independence) and 7 represented extremely positive feelings (i.e., easy to use, helpful, simple, more independence).

Individuals were asked to indicate how they felt about each specific feature of the smartphone and health record on a scale from 1 (terrible) to 7 (delighted). These features included: having their own personal health record; medication prompts they receive; the appointment/schedule prompts they receive; connecting with their care provider using the smartphone; connecting with their care provider using the LSR; having access to their personal crisis plan; and being able to share their health information with other health care providers.

Participants were asked specifically whether or not they used the LSR, and whether or not they used the smartphone in order to determine utilization rates. They were also asked to indicate what they used the smartphone for. The list of possible uses included accessing the LSR, contacting their care provider, use of social media (e.g., Facebook, Twitter), texting, emailing, playing games, listening to music, watching videos, or other. If they indicated “other” they were asked to give specific details of use. For the current analysis, these details were examined and additional categories created. Each participant could indicate multiple items."
6a-i) Online questionnaires: describe if they were validated for online use and apply CHERRIES items to describe how the questionnaires were designed/deployed

6a-ii) Describe whether and how “use” (including intensity of use/dosage) was defined/measured/monitored

6a-iii) Describe whether, how, and when qualitative feedback from participants was obtained

6b) CONSORT: Any changes to trial outcomes after the trial commenced, with reasons
"Most items from the Perception of SMART Technology questionnaire were collected at the six, twelve, and eighteen month interviews. The exceptions were the questions on feelings about connecting with their care provider through their health record, questions on utilization of the smartphone and LSR, and what participants were using their smartphones for. This information was only collected at the twelve and eighteen month interviews."

Questions were added to the survey after the 6 month interview because the research team determined this information would be useful in understanding how clients were using the technology and what their perceptions of the technology were.
7a) CONSORT: How sample size was determined
7a-i) Describe whether and how expected attrition was taken into account when calculating the sample size

7b) CONSORT: When applicable, explanation of any interim analyses and stopping guidelines

Not applicable. No interim analyses were completed and no stopping guidelines were in place, though participants could stop using the technologies at any point they chose.

8a) CONSORT: Method used to generate the random allocation sequence

Clients were allocated to Group A and Group B in an alternating fashion as care providers enrolled participants in the study.

"Community-based individuals from the caseloads of participating mental health care professionals were randomized into two groups: individuals in Group A (early intervention group) received the smart technology intervention first, while those in Group B (delayed intervention group) acted as a control for the first six months, and thus received the intervention six months after Group A."

"As there were no significant differences between the two intervention groups post-randomization, they were collapsed into one group for purposes of the current analysis."

8b) CONSORT: Type of randomisation; details of any restriction (such as blocking and block size)

Not applicable.

9) CONSORT: Mechanism used to implement the random allocation sequence (such as sequentially numbered containers), describing any steps taken to conceal the sequence until interventions were assigned

Not applicable.

10) CONSORT: Who generated the random allocation sequence, who enrolled participants, and who assigned participants to interventions

Care providers enrolled participants in the study and the research team assigned participants to intervention groups.

11a) CONSORT: Blinding - If done, who was blinded after assignment to interventions (for example, participants, care providers, those assessing outcomes) and how

11a-i) Specify who was blinded, and who wasn't

Not applicable. Participants, care providers, and researchers were not blinded to intervention group.

11a-ii) Discuss e.g., whether participants knew which intervention was the "intervention of interest" and which one was the "comparator"

11b) CONSORT: If relevant, description of the similarity of interventions

Not applicable. All participants eventually received the same intervention.

12a) CONSORT: Statistical methods used to compare groups for primary and secondary outcomes

"Frequencies and percentages were calculated for all categorical data (e.g., sample characteristics, baseline comfort with technology, utilization of the smart technologies) and means and standard deviations were calculated for all scale variables (e.g., feelings towards the technologies in general and the specific features of each). Paired t-tests were used to determine differences between six and twelve month post intervention data for perceptions of the smartphone and LSR regarding ease of use, helpfulness, simplicity, and independence they afforded. Perceptions about specific features of the smartphone and LSR were also compared. Additionally, a paired t-test was used to examine whether differences existed between perceptions of the smartphone and the health record at twelve months post intervention. Each specific analysis was conducted on a complete case basis and all data analyses were done using Statistical Package for Social Sciences (SPSS) 22.0."

12a-i) Imputation techniques to deal with attrition / missing values

Not applicable. Data was analyzed on a complete case basis.

12b) CONSORT: Methods for additional analyses, such as subgroup analyses and adjusted analyses

Not applicable. No additional analyses were completed.

RESULTS

13a) CONSORT: For each group, the numbers of participants who were randomly assigned, received intended treatment, and were analysed for the primary outcome

"As a result of dropouts and loss of contact with participants, the analysis presented here is based on 394 individuals who completed the study. Group A consisted of 192 individuals and Group B consisted of 202 individuals."

13b) CONSORT: For each group, losses and exclusions after randomisation, together with reasons

"In total, 400 community based participants were recruited from the caseloads of 54 mental health care professionals in London, Ontario and the surrounding area... As a result of dropouts and loss of contact with participants, the analysis presented here is based on 394 individuals who completed the study. Group A consisted of 192 individuals and Group B consisted of 202 individuals."

13b-i) Attrition diagram

14a) CONSORT: Dates defining the periods of recruitment and follow-up

"The project began in September of 2011 and was completed in March 2014 within London, Ontario, Canada and the surrounding area."

14a-i) Indicate if critical "secular events" fell into the study period

14b) CONSORT: Why the trial ended or was stopped (early)

Not applicable. The trial was not ended or stopped early.

15) CONSORT: A table showing baseline demographic and clinical characteristics for each group

"The average age of MHEN participants was 37.6 years, and the majority of participants were male (60.7%) and/or were single and had never been married (70.1%). Just under half (45.0%) of the individuals in the study had graduated high school and almost a quarter (24.7%) had completed post-secondary schooling. The most prevalent psychiatric diagnosis in the sample was a psychotic disorder (59.4%) followed closely by a mood disorder (57.4%). The least prevalent diagnoses were personality disorder (6.1%), disorder of childhood/adolescence (5.6%), and other/organic/unknown type (4.8%). No significant differences between the early intervention group and delayed intervention were found on any baseline demographics (Table 1)."

15-i) Report demographics associated with digital divide issues

Not applicable. Digital divide issues were not reported in this study.

16a) CONSORT: For each group, number of participants (denominator) included in each analysis and whether the analysis was by original assigned groups

16-i) Report multiple "denominators" and provide definitions

"As there were no significant differences between the two intervention groups post-randomization, they were collapsed into one group for purposes of the current analysis."

"As a result of dropouts and loss of contact with participants, the analysis presented here is based on 394 individuals who completed the study."

16-ii) Primary analysis should be intent-to-treat

17a) CONSORT: For each primary and secondary outcome, results for each group, and the estimated effect size and its precision (such as 95% confidence interval)

"At the outset of the study, the majority of participants felt comfortable with all of the technologies that were investigated (computers, phone, and technology in general; Figure 2). Almost the entire sample (n=362, 91.9%) felt comfortable with phones, and approximately two-thirds felt comfortable with computers (n=267, 68.1%) and technology in general (n=277, 70.3%). Alternatively, only a small fraction (n=16, 4.1%) felt uncomfortable with phones, and less than one-fifth of the sample felt uncomfortable with computers (n=70, 17.9%) or technology in general (n=55, 14.0%)."

"Given a neutral score of four (half way between the extreme negative and positive scores) it appears perceptions of ease of use, helpfulness, simplicity, and provision of independence for both the smartphone and the LSR were generally positive as all averaged scores ranged from 4.83 to 6.29 (Table 2). Only two aspects significantly changed over time: positive perceptions towards the smartphone's ease of use increased between 6 and 12 months post intervention ($t=-3.112$, $p = 0.002$); while positive perceptions towards the LSR's helpfulness decreased ($t=4.443$, $p < 0.001$)."

"When comparing the smartphone and LSR at 12 months post intervention, individuals consistently rated the smartphone higher than the LSR. The difference in average scores between the two technologies ranged from 0.56 (simplicity) to 1.01 (ease of use), depending on the utility being examined, and were all significant (all $P < 0.001$; Table 3)."

"Again, perceptions of specific functions of the smartphone and the LSR tended to be positive overall, with scores ranging from 4.97 to 5.90 (Table 4). Over time there was a significant decrease in the positivity of perceptions towards having the LSR (difference of 0.24, $P = 0.002$), and having access to a personal crisis plan (difference of 0.29, $P = 0.009$). No other significant changes were found."

"The five most common uses of the smartphone were related to communication (Table 5). Of those who indicated they were currently using the smartphone, 247 (79.4%) indicated they were using it to send and receive text messages, 240 (77.2%) indicated they were using it to contact their care provider and 205 (65.9%) indicated they were using it to send and receive email messages. Accessing the LSR was the seventh most common activity with 145 (46.6%) of those reporting on the use of the smartphone."

"At the study's completion a total of 62 devices had been lost, sold, broken, stolen, or permanently locked at some point during the study. Of the 30 devices that had been lost, 10 were later found by participants, resulting in a total of 52 unusable or misplaced devices. Of these, 20 (38.5%) had been lost, 17 (32.7%) had been stolen, 8 (15.3%) had been broken, 6 (11.5%) had been sold, and 1 (1.9%) had been permanently locked due to the security features of the operating system."

17a-i) Presentation of process outcomes such as metrics of use and intensity of use

17b) CONSORT: For binary outcomes, presentation of both absolute and relative effect sizes is recommended
"At 12 months post intervention 311 (93.4%) of the participants who answered questions about use of the smartphone and PHR indicated they were currently using the smartphone. In contrast, 151 (45.3%) indicated they were currently using the LSR."
18) CONSORT: Results of any other analyses performed, including subgroup analyses and adjusted analyses, distinguishing pre-specified from exploratory
Not applicable. No other analyses were performed.
18-i) Subgroup analysis of comparing only users

19) CONSORT: All important harms or unintended effects in each group
No harms occurred. The only unintended effect was when clients found the LSR's functions insufficient, they independently programmed similar functions into the smartphone itself: "However, participants often found these functions within the LSR to be lacking, and consequently used functions native to the smartphone, such as the calendar, for these purposes. These findings suggest that while the smartphone and its functions appear to be helpful, the LSR will benefit from further modification."
19-i) Include privacy breaches, technical problems

19-ii) Include qualitative feedback from participants or observations from staff/researchers

DISCUSSION

20) CONSORT: Trial limitations, addressing sources of potential bias, imprecision, multiplicity of analyses
20-i) Typical limitations in ehealth trials
"For example, since the LSR is often accessed using the smartphone, it is difficult to evaluate the phone and the record as separate entities. While the LSR is available in both desktop computer and mobile phone versions, it is possible that clients were using their smartphones to access the version of the LSR designed for use on desktop computers. It is important to understand how individuals prefer to access their information and what characteristics make a function appealing and usable. Fully understanding user preferences may not be possible without knowing the way in which they accessed the LSR, but this was not probed in the present study. Another possible limitation is the link between a client's perception of the intervention and their care provider's willingness or ability to use the intervention. The intervention examined in the MHEN study involved two-way input from clients and care providers. In order for clients to perceive the intervention positively, care providers must be able to support clients in the technical aspects of the intervention and also be actively engaged in the intervention themselves [33]. Unfortunately, active care provider participation was not always present and for this reason, some clients may have perceived the LSR negatively."
21) CONSORT: Generalisability (external validity, applicability) of the trial findings
21-i) Generalizability to other populations

21-ii) Discuss if there were elements in the RCT that would be different in a routine application setting

22) CONSORT: Interpretation consistent with results, balancing benefits and harms, and considering other relevant evidence
22-i) Restate study questions and summarize the answers suggested by the data, starting with primary outcomes and process outcomes (use)
"The MHEN project used mobile phones and web based technologies to support the care of individuals experiencing mental illness [8-10]. The purpose of this secondary analysis was to determine how participants in the study perceived the use of the smartphone and the PHR in their mental health care and in their lives generally... At baseline, individuals indicated that they were generally comfortable using technology...Participants perceived the LSR and mobile devices used in this intervention positively in terms of ease of use, helpfulness, simplicity, and the independence they afforded...Overall, participants rated specific functions of the technologies, such as appointment reminders, as being perceived positively. Participants' perceptions of having a PHR and having access to a personal crisis plan decreased over time. Other functions, such as medication and appointment reminders, proved to be important to many participants...Consistent with previous research findings [12], participants most commonly reported using the smartphone for communication...A concern at the outset of the study was maintenance and retention of devices. The small percentage of devices that were lost, stolen, broken, or inactive at the completion of the study provides evidence that individuals diagnosed with severe mental illnesses are accountable to manage and maintain devices for personal and health management purposes."
22-ii) Highlight unanswered new questions, suggest future research

Other information

23) CONSORT: Registration number and name of trial registry

"The Mental Health Engagement Network (MHEN; Trial Registration: ClinicalTrials.gov NCT01473550) project used web and mobile technologies to distribute and evaluate the use of a Personal Health Record (PHR) to assist mental health clients in their care [8-10]."

24) CONSORT: Where the full trial protocol can be accessed, if available

Not available.

25) CONSORT: Sources of funding and other support (such as supply of drugs), role of funders

"This research was supported by funds from Canada Health Infoway. TELUS provided telecommunication and infrastructure support."

X26-i) Comment on ethics committee approval

x26-ii) Outline informed consent procedures

X26-iii) Safety and security procedures

X27-i) State the relation of the study team towards the system being evaluated
